# Supplementary material for: Vulnerabilities in coronavirus glycan shields despite extensive glycosylation
Source: Nat Commun. 2020 May 27;11:2688. doi: 10.1038/s41467-020-16567-0 (PMC7253482; doi:10.1038/s41467-020-16567-0)
Supplement: Supplementary file 1 — Supplementary Information [file 41467_2020_16567_MOESM1_ESM.pdf]

Supplementary Information for:

Vulnerabilities in coronavirus glycan shields despite extensive glycosylation

Yasunori Watanabe[1,2,3], Zachary T. Berndsen[4], Jayna Raghwani[5], Gemma E. Seabright[1,2], Joel D. Allen[1], Oliver G. Pybus[8], Jason S. McLellan[7], Ian A. Wilson[4,6], Thomas A. Bowden[3], Andrew B. Ward[4], Max Crispin[1]*

[1] School of Biological Sciences, University of Southampton, Southampton, SO17 1BJ, UK

[2] Oxford Glycobiology Institute, Department of Biochemistry, University of Oxford, South Parks Road, Oxford OX1 3QU, UK

[3] Division of Structural Biology, University of Oxford, Wellcome Centre for Human Genetics, Oxford OX3 7BN, UK

[4] Department of Integrative Structural and Computational Biology, The Scripps Research Institute, La Jolla, CA 92037, USA

[5] Big Data Institute, Li Ka Shing Centre for Health Information and Discovery, Nuffield Department of Medicine, University of Oxford, Oxford, OX3 7LF, UK

[6] Skaggs Institute for Chemical Biology, The Scripps Research Institute, La Jolla, CA 92037, USA

[7] Department of Molecular Biosciences, The University of Texas at Austin, Austin, TX 78712, USA.

[8] Department of Zoology, University of Oxford, Oxford, OX1 3PS, UK

*To whom correspondence may be addressed. Email: max.crispin@soton.ac.uk

This document includes Supplementary Figures 1-7, Supplementary Figure Legends, and Supplementary References.

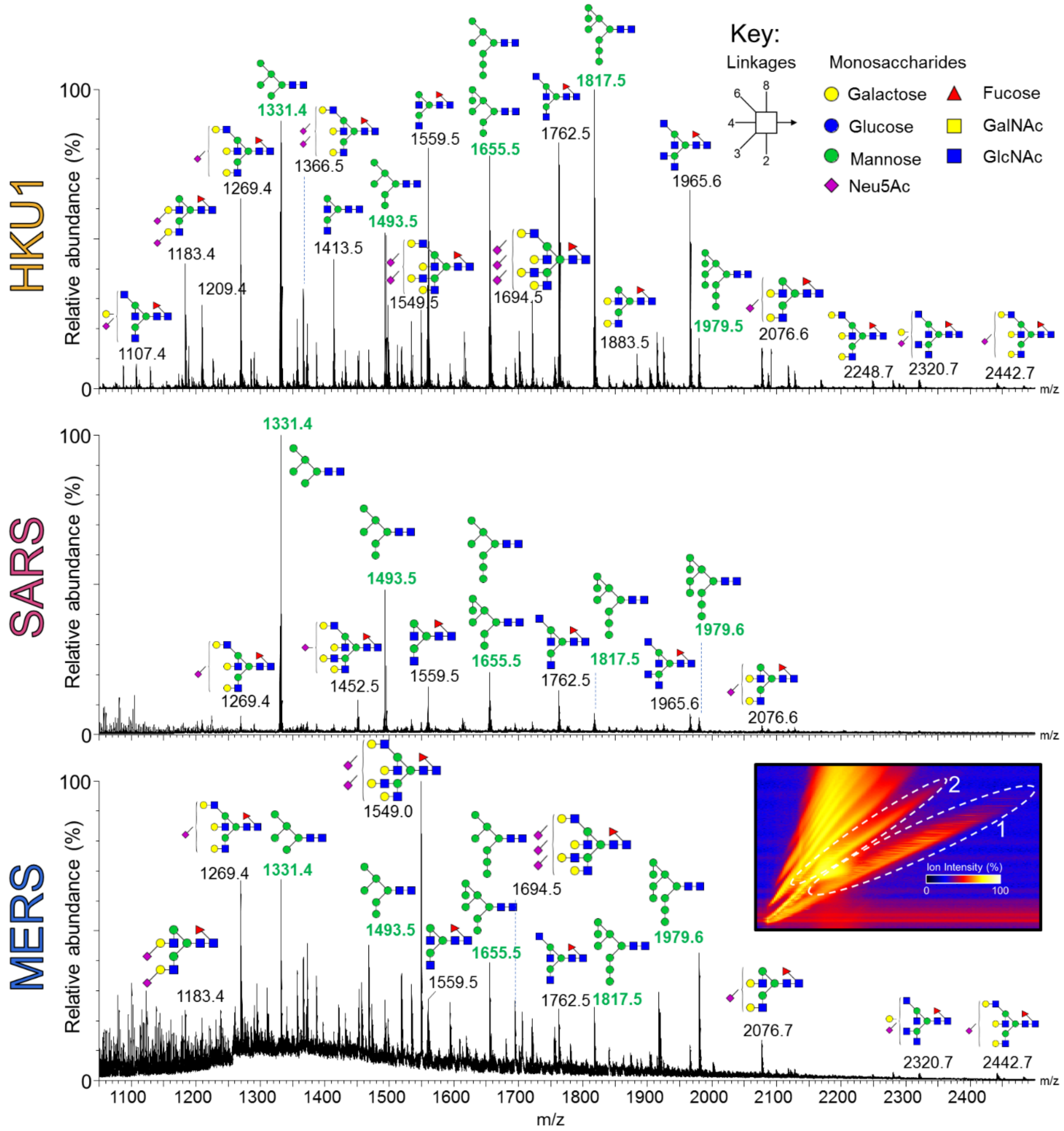

Fig. S1. Ion mobility-extracted mass spectra of singly- and doubly- charged N-linked glycan ions from HKU1, SARS and MERS S glycoproteins. Peaks are annotated with the corresponding compositions, using Consortium for Functional Glycomics symbolic nomenclature and Oxford system linkages[1], as per the key. Oligomannose-type glycan *m/z* values are labelled in green.

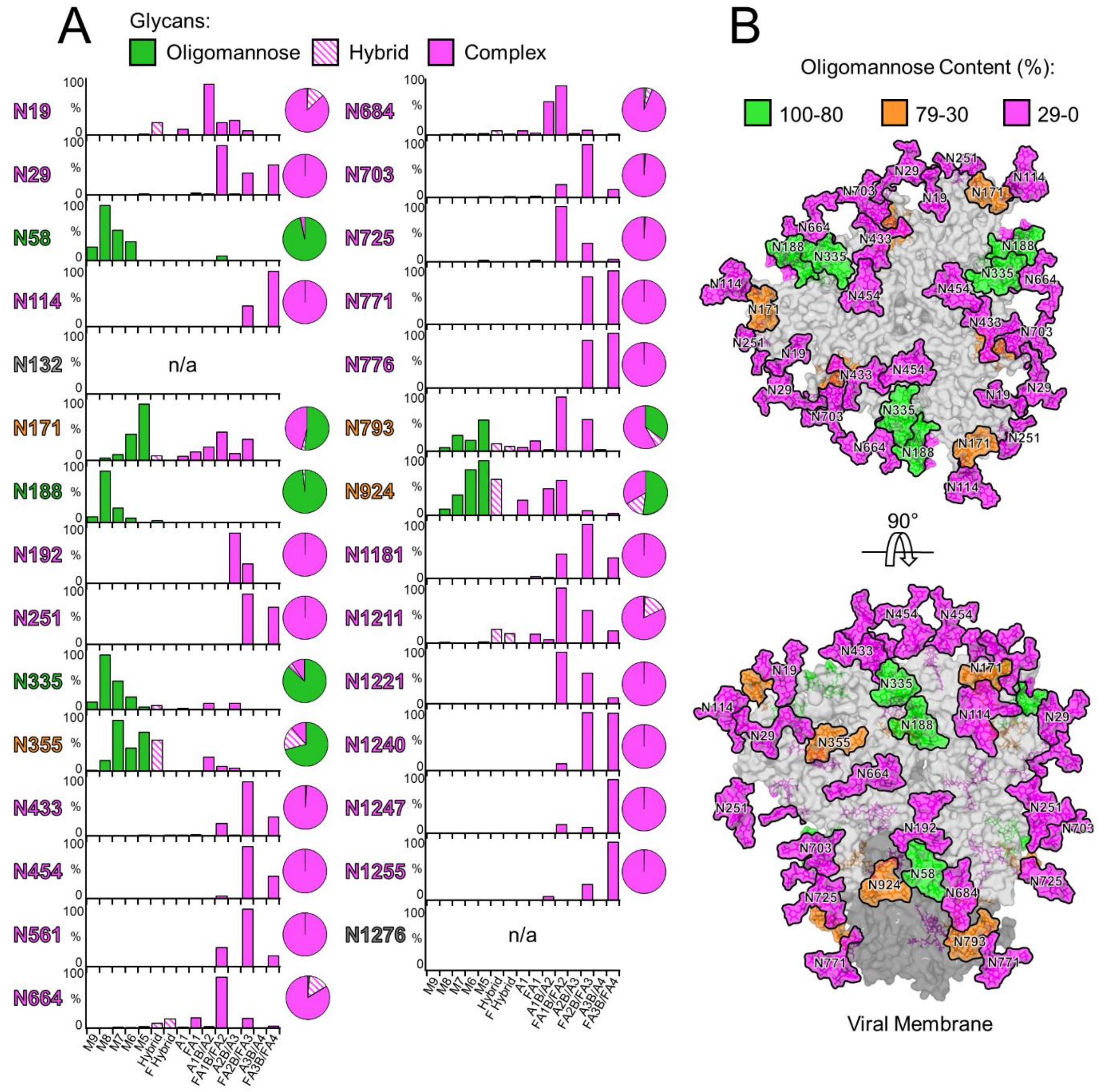

Fig. S2. Compositional analysis and structure-based mapping of HKU1 S N-linked glycans. (A) Quantitative site-specific N-linked analysis of HKU1 S. Purified HKU1 S was digested with trypsin, chymotrypsin, and trypsin + chymotrypsin, then analysed by LC-ESI MS. Glycan compositions are based on the glycan library generated from negative-ion mass spectrometry of released N-glycans. The bar graphs represent the relative quantities of each glycan group with oligomannose-type glycan series (M9 to M5; Man$_9$GlcNAc$_2$ to Man$_5$GlcNAc$_2$) (green), afucosylated and fucosylated hybrid glycans (Hybrid & F Hybrid) (dashed pink), and complex glycans grouped according to the number of antennae and fucosylation (A1 to FA4) (pink). Left to right; least-processed to most processed. The pie charts summarise the quantification of these glycans. (B) Modelling of experimentally observed glycosylation onto the pre-fusion structure of trimeric HKU1 S (PDB ID code 5I08)[2]. The glycans are coloured according to

oligomannose content, as defined by the upper right-hand key. S1 and S2 subunits coloured light grey and dark grey, respectively.

MERS	N66	N104	N125	N155	N166	N222	N236	N244	N410	N487	N592	N619	N719	N774	N785	N870	N1176	N1213	N1225	N1241	N1256	N1277	N1288
Fucosylation (%)	0	0	0	0	0	0	3	0	0	100	100	100	100	99	99	26	100	19	100	100	100	100	0
Sialylation(%)	0	18	0	0	0	0	0	28	0	25	38	22	30	31	27	19	66	0	0	7	0	0	0

SARS	N29	N65	N73	N109	N118	N119	N158	N227	N269	N318	N330	N357	N589	N602	N691	N699	N783	N1056	N1080	N1116	N1140	N1155	N1176
Fucosylation (%)	97	0	91	98	99	99	0	0	75	98	97	77	0	64	98	51	5	83	12	100	96	100	97
Sialylation(%)	18	11	17	22	54	69	0	0	22	32	28	68	0	14	3	99	1	16	36	0	4	0	35

HKU1	N19	N29	N58	N114	N132	N171	N188	N192	N251	N335	N355	N433	N454	N561	N664	N684	N703	N725	N771	N776	N793	N924	N1181	N1211	N1221	N1240	N1255	N1276
Fucosylation (%)	17	100	3	100	n/a	33	0	48	100	0	2	99	100	100	92	57	99	99	100	100	58	16	99	87	100	100	96	n/a
Sialylation(%)	8	16	0	0	n/a	0	0	48	0	0	0	8	0	13	29	31	11	23	0	0	3	1	1	8	53	0	0	n/a

SI Fig 3. Site-specific quantification of fucosylation and sialylation of N-linked glycan sites, on MERS, SARS, and HKU1 S glycoproteins.

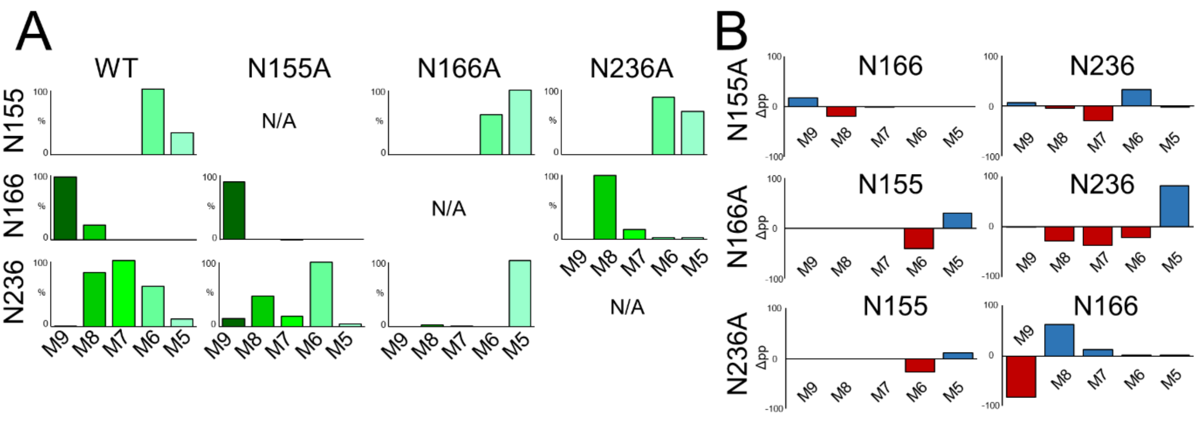

SI Fig 4. Glycan deletion increases mannose trimming of N-linked glycans on MERS S oligomannose patch. (A) Relative quantitation of glycans in the mannose patch sites (N155, N166, N236) on MERS S. M9 to M5; ($Man_9GlcNAc_2$ to $Man_5GlcNAc_2$) (dark green to pale green). (B) Percentage point differences in the abundance of oligomannose-type glycans at mannose patch sites in the glycan knockout mutants compared to WT MERS. Decreased and increased abundances are colored red and blue, respectively.

	MERS S	SARS S	HKU1 S	LASV GPC[3]	HIV-1 BG505 Env[4]	SIV MT145K Env[5]	H3N2 Vic11 HA
Oligomannose-type (%)	33.8	32.2	25.0	49.5	63.0	70.5	50.1
Complex-type (%)	66.2	67.8	75.0	50.5	37.0	29.5	49.9

SI Fig. 5 Oligomannose- and complex-type glycan composition table of viral fusion proteins, quantified by HILIC-UPLC. Endo H digestions of labelled glycans were performed to measure oligomannose abundance.

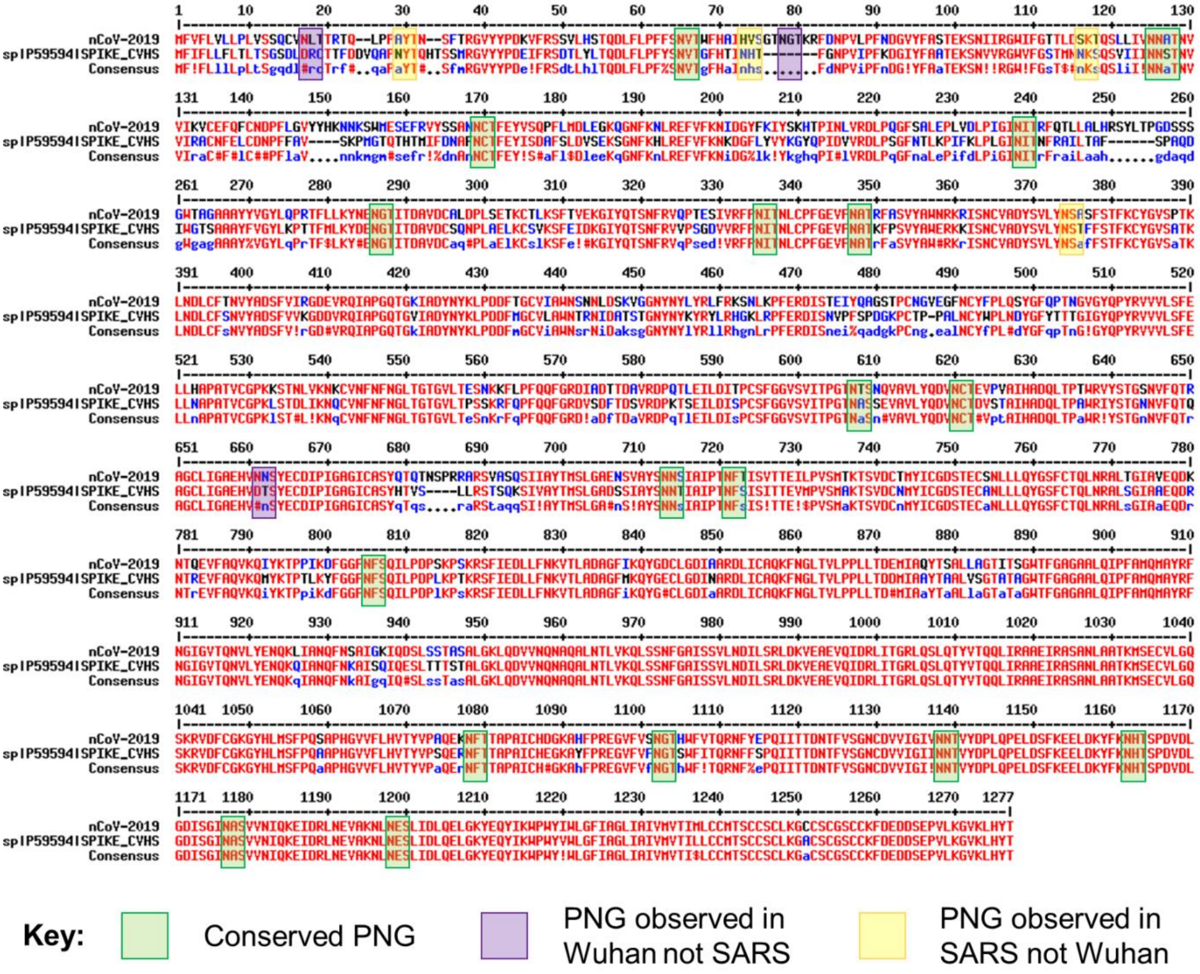

SI Fig. 6: Sequence alignment of S proteins SARS-CoV-2 (Genbank: MN908947.3) and SARS CoV (Uniprot: P59594) highlighting conservation of N-linked glycan sequons. Conserved potential N-linked glycosylation sites (PNGs) are colored in green, PNGs observed in SARS-CoV-2 but not in SARS are colored purple, and PNGs observed in SARS but not SARS-CoV-2 are colored in yellow.

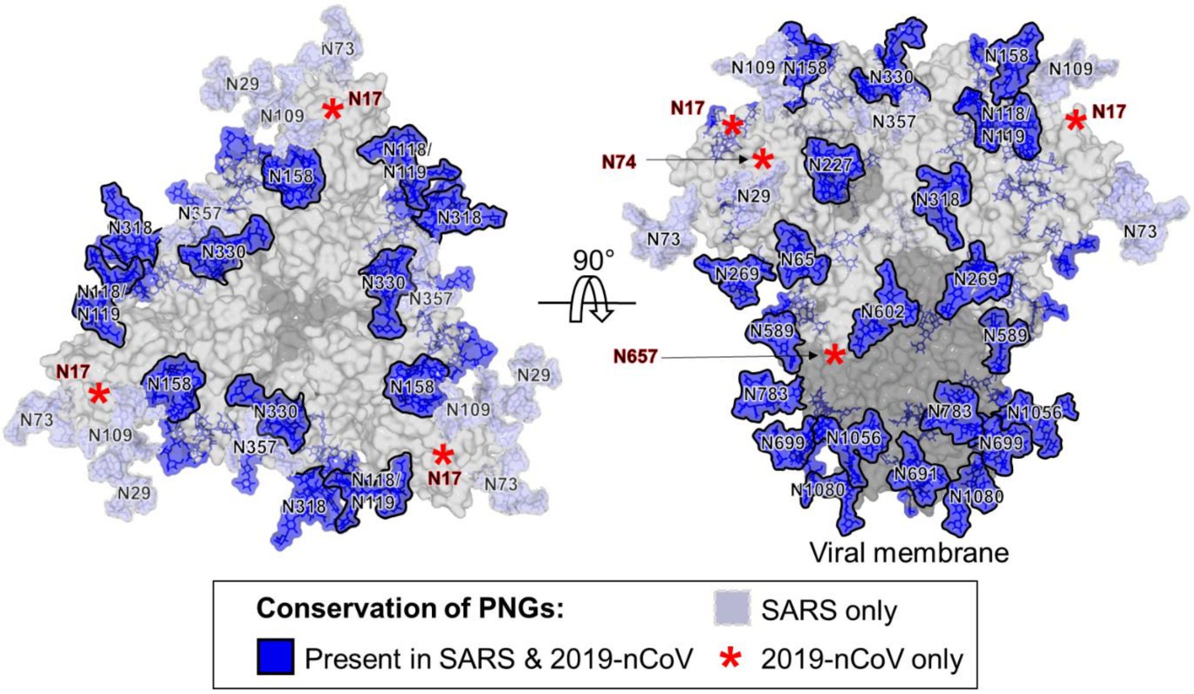

SI Fig 7: Mapping the conservation of glycosylation sites between SARS and SARS-CoV-2. Glycan sites were modelled onto SARS S (PDB ID:5X58)[6], with glycan sites conserved between both viruses, colored blue. Glycan sites only present in SARS are colored in light blue-grey. Approximate positions of N-linked glycans present on SARS-CoV-2 are highlighted by red asterisks, with numbering based on the SARS-CoV-2 protein sequence.

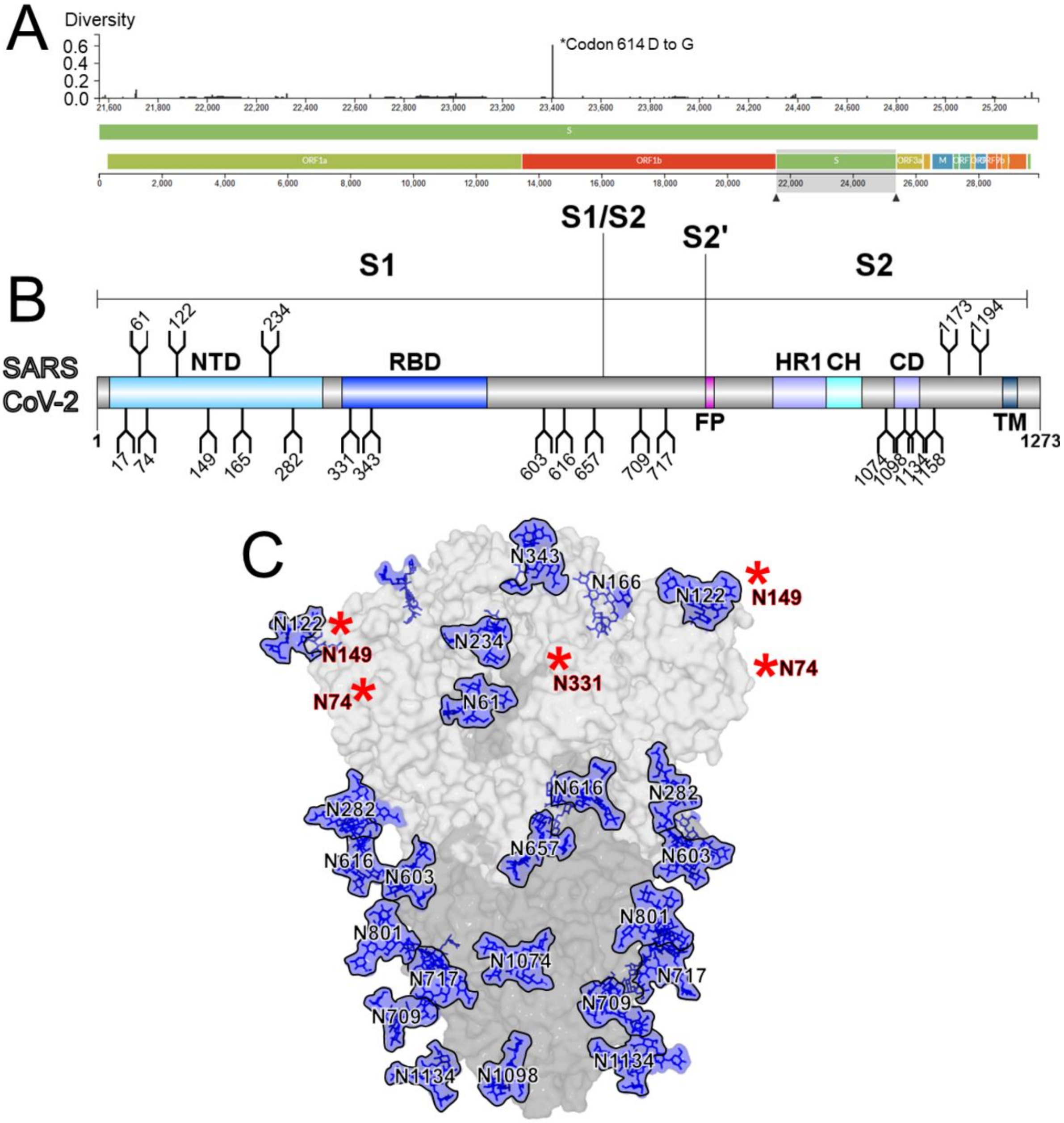

SI Fig 8: (A) Low genetic diversity and conservation of SARS-CoV-2 N-linked glycosylation sites. Sequence diversity of S gene taken from nextstrain (17th March 2020, n=566)[7] (https://nextstrain.org/ncov). The single spike corresponds to codon position 614 which changes from D to G with ~50% frequency. Bioinformatic analysis revealed no differences in predicted N-linked glycosylation sites within these 566 strains. **(B)** Schematic representation of SARS-CoV-2 S glycoprotein, showing the positions of N-linked glycosylation amino-acid sequons (NXS/T, where X ≠ P) shown as branches. The domains of the S glycoproteins are illustrated: N-terminal domain (NTD), receptor-binding domain (RBD), fusion peptide (FP), heptad repeat 1 (HR1), central helix (CH), connector domain (CD) and transmembrane domain (TM), as characterised by Wrapp et al. [8] **(C)** A fully glycosylated model of SARS-CoV-2 S protein (PDB ID: 6VSB) with the three receptor-binding domains in the "down" conformation. $Man_5GlcNAc_2$ glycan compositions were modelled at each site. Note that the positions of glycosylation sites at N74, N149, and N331, which reside on extended loops that have not been

structurally resolved, are annotated by red astericks. There are also N-linked glycosylation sites at N17, N1158, N1173, and N1194 that are not structurally resolved.

Glycan library:

HexNAc(2)Hex(3), HexNAc(2)Hex(4), HexNAc(2)Hex(4)Fuc(1), HexNAc(2)Hex(5), HexNAc(2)Hex(6), HexNAc(2)Hex(7), HexNAc(2)Hex(8), HexNAc(2)Hex(9), HexNAc(3)Hex(3), HexNAc(3)Hex(3)Fuc(1), HexNAc(3)Hex(4), HexNAc(3)Hex(4)Fuc(1), HexNAc(3)Hex(4)Fuc(1)NeuAc(1), HexNAc(3)Hex(5), HexNAc(3)Hex(5)NeuAc(1), HexNAc(3)Hex(5)Fuc(1), HexNAc(3)Hex(5)Fuc(1)NeuAc(1), HexNAc(3)Hex(6), HexNAc(3)Hex(6)NeuAc(1), HexNAc(3)Hex(6)Fuc(1), HexNAc(3)Hex(6)Fuc(1)NeuAc(1), HexNAc(4)Hex(3), HexNAc(4)Hex(3)Fuc(1), HexNAc(4)Hex(4), HexNAc(4)Hex(4)Fuc(1), HexNAc(4)Hex(4)Fuc(1)NeuAc(1), HexNAc(4)Hex(4)Fuc(2), HexNAc(4)Hex(5),

HexNAc(4)Hex(5)NeuAc(1), HexNAc(4)Hex(5)Fuc(1), HexNAc(4)Hex(5)Fuc(1)NeuAc(1), HexNAc(4)Hex(5)Fuc(1)NeuAc(2), HexNAc(4)Hex(5)Fuc(2), HexNAc(4)Hex(5)Fuc(3), HexNAc(5)Hex(3), HexNAc(5)Hex(3)Fuc(1), HexNAc(5)Hex(3)Fuc(2), HexNAc(5)Hex(4)NeuAc(1), HexNAc(5)Hex(4)Fuc(1), HexNAc(5)Hex(4)Fuc(1)NeuAc(1), HexNAc(5)Hex(4)Fuc(2) , HexNAc(5)Hex(4)Fuc(2), HexNAc(5)Hex(4)Fuc(2)NeuAc(1), HexNAc(5)Hex(4)Fuc(3),

HexNAc(5)Hex(5)Fuc(1), HexNAc(5)Hex(5)Fuc(1)NeuAc(1), HexNAc(5)Hex(5)Fuc(2), HexNAc(5)Hex(6)NeuAc(1), HexNAc(5)Hex(6)Fuc(1), HexNAc(5)Hex(6)Fuc(1)NeuAc(1), HexNAc(5)Hex(6)Fuc(1)NeuAc(2), HexNAc(5)Hex(6)Fuc(1)NeuAc(3), HexNAc(5)Hex(6)Fuc(2), HexNAc(6)Hex(3)Fuc(1),

HexNAc(6)Hex(3)Fuc(1)NeuAc(1), HexNAc(6)Hex(3)Fuc(2), HexNAc(6)Hex(3)Fuc(3), HexNAc(6)Hex(4)Fuc(1), HexNAc(6)Hex(4)Fuc(2), HexNAc(6)Hex(5)Fuc(1), HexNAc(6)Hex(5)Fuc(1)NeuAc(2), HexNAc(6)Hex(6)Fuc(1)NeuAc(3), HexNAc(6)Hex(7)Fuc(1)NeuAc(2), HexNAc(6)Hex(7)Fuc(1)NeuAc(3), HexNAc(6)Hex(7)Fuc(1)NeuAc(4), HexNAc(7)Hex(3)Fuc(1), HexNAc(7)Hex(4), HexNAc(7)Hex(4)Fuc(1)

SI Fig. 9. Glycan library generated from ESI-IM MS used as post-translational modifications for mass spectrometry of glycopeptides.

Supplementary References

1. Harvey, D. J. *et al.* Proposal for a standard system for drawing structural diagrams of N- and O-linked carbohydrates and related compounds. *Proteomics* **9**, 3796–3801 (2009).

2. Kirchdoerfer, R. N. *et al.* Pre-fusion structure of a human coronavirus spike protein. *Nature* **531**, 118–121 (2016).

3. Watanabe, Y. *et al.* Structure of the Lassa virus glycan shield provides a model for

immunological resistance. *Proc. Natl. Acad. Sci.* **115**, 7320–7325 (2018).

4. Behrens, A.-J. *et al.* Composition and antigenic effects of individual glycan sites of a trimeric HIV-1 envelope glycoprotein. *Cell Rep.* **14**, 2695–2706 (2016).

5. Andrabi, R. *et al.* The chimpanzee SIV envelope trimer: structure and deployment as an HIV vaccine template. *Cell Rep.* **27**, 2426-2441.e6 (2019).

6. Yuan, Y. *et al.* Cryo-EM structures of MERS-CoV and SARS-CoV spike glycoproteins reveal the dynamic receptor binding domains. *Nat. Commun.* **8**, 15092 (2017).

7. Hadfield, J. *et al.* Nextstrain: real-time tracking of pathogen evolution. *Bioinformatics* **34**, 4121–4123 (2018).

8. Wrapp, D. *et al.* Cryo-EM structure of the 2019-nCoV spike in the prefusion conformation. *Science* (2020). doi:10.1126/science.abb2507
