# Supplementary material for: Vulnerabilities in coronavirus glycan shields despite extensive glycosylation
Source: Nat Commun. 2020 May 27;11:2688. doi: 10.1038/s41467-020-16567-0 (PMC7253482; doi:10.1038/s41467-020-16567-0)
Supplement: Supplementary file 2 — Reporting Summary [file 41467_2020_16567_MOESM2_ESM.pdf]

nature research

Corresponding author(s): Max Crispin

Last updated by author(s): Apr 27, 2020

Reporting Summary

Nature Research wishes to improve the reproducibility of the work that we publish. This form provides structure for consistency and transparency in reporting. For further information on Nature Research policies, see Authors & Referees and the Editorial Policy Checklist.

Statistics

For all statistical analyses, confirm that the following items are present in the figure legend, table legend, main text, or Methods section.

n/a	Confirmed	
☐	☒	The exact sample size (*n*) for each experimental group/condition, given as a discrete number and unit of measurement
☐	☒	A statement on whether measurements were taken from distinct samples or whether the same sample was measured repeatedly
☒	☐	The statistical test(s) used AND whether they are one- or two-sided<br>*Only common tests should be described solely by name; describe more complex techniques in the Methods section.*
☒	☐	A description of all covariates tested
☒	☐	A description of any assumptions or corrections, such as tests of normality and adjustment for multiple comparisons
☐	☒	A full description of the statistical parameters including central tendency (e.g. means) or other basic estimates (e.g. regression coefficient) AND variation (e.g. standard deviation) or associated estimates of uncertainty (e.g. confidence intervals)
☒	☐	For null hypothesis testing, the test statistic (e.g. *F*, *t*, *r*) with confidence intervals, effect sizes, degrees of freedom and *P* value noted<br>*Give P values as exact values whenever suitable.*
☐	☒	For Bayesian analysis, information on the choice of priors and Markov chain Monte Carlo settings
☒	☐	For hierarchical and complex designs, identification of the appropriate level for tests and full reporting of outcomes
☒	☐	Estimates of effect sizes (e.g. Cohen's *d*, Pearson's *r*), indicating how they were calculated

Our web collection on statistics for biologists contains articles on many of the points above.

Software and code

Policy information about availability of computer code

Data collection	Mass spectrometry: MassLynx v4.11, Orbitrap tune software v3.1, Xcalibur v4.2
Data analysis	Mass spectrometry: MassLynx v4.11 and Driftscope version 2.8 software; ByonicTM (Version 3.5.0) and ByologicTM (Version 3.5-15; Protein Metrics Inc.)<br>UPLC analysis: Empower 3 software (Waters)<br>EM analysis: EM movie micrographs were aligned and dose weighted with MotionCor2 and CTF estimation was perfomred with Gctf. Maps were low-pass filtered using a Gaussian kernel and visualised in UCXXF chimera.<br>A detailed description of the auto-thresholoding method used to set the isosurface value for visualisation of low-pass filtered maps can be found in the cited Berndsen et al. (CryoSparc v.2, Relion v.3, SPARX)<br>Evolutionary analysis: BEAST v1.8.4

For manuscripts utilizing custom algorithms or software that are central to the research but not yet described in published literature, software must be made available to editors/reviewers. We strongly encourage code deposition in a community repository (e.g. GitHub). See the Nature Research guidelines for submitting code & software for further information.

Data

Policy information about availability of data

All manuscripts must include a data availability statement. This statement should provide the following information, where applicable:

- Accession codes, unique identifiers, or web links for publicly available datasets
- A list of figures that have associated raw data
- A description of any restrictions on data availability

All data is available upon request. In addition to the data in this paper and accompanying supplementary materials, the raw mass spectrometric data have been deposited on the MassIVE server (http://massive.ucsd.edu) with the accession codes (MSV000084993 for glycopeptide analysis, and MSV000085182 for N-linked glycans).

Field-specific reporting

Please select the one below that is the best fit for your research. If you are not sure, read the appropriate sections before making your selection.

☒ Life sciences ☐ Behavioural & social sciences ☐ Ecological, evolutionary & environmental sciences

For a reference copy of the document with all sections, see nature.com/documents/nr-reporting-summary-flat.pdf

Life sciences study design

All studies must disclose on these points even when the disclosure is negative.

Sample size	3. In this study we analysed three pre-selected coronavirus glycoproteins
Data exclusions	Mass spectrometric analysis requires an accuracy cut-off that was pre-established and which we describe in the paper, 4ppm for precursor ions and 10 ppm for fragment ions. These parameters were chosen as the Orbitrap instruent is calibrated to 2ppm, thus provides 2ppm leeway but also minimizes false positive assignments.
Replication	Identity of glycans were supported by using three different proteases giving different pools of glycopeptides which confirmed assignments
Randomization	N/A. Randomisation is not relevant to this study, since we are studying the glycosylation of selected coronavirus spike proteins.
Blinding	N/A. Site-specific analysis of single samples where sequence idenitity is revealed during analysis prevents blinding. However, the use of analysis with predetermined parameters limits user bias.

Reporting for specific materials, systems and methods

We require information from authors about some types of materials, experimental systems and methods used in many studies. Here, indicate whether each material, system or method listed is relevant to your study. If you are not sure if a list item applies to your research, read the appropriate section before selecting a response.

Materials & experimental systems

n/a	Involved in the study	
☒	☐	Antibodies
☐	☒	Eukaryotic cell lines
☒	☐	Palaeontology
☒	☐	Animals and other organisms
☒	☐	Human research participants
☒	☐	Clinical data

Methods

n/a	Involved in the study	
☒	☐	ChIP-seq
☐	☐	Flow cytometry
☒	☐	MRI-based neuroimaging

Eukaryotic cell lines

Policy information about cell lines

Cell line source(s)	FreeStyleTM 293-F cells sourced from ThermoFisher Scientific
Authentication	Cell lines were not authenticated
Mycoplasma contamination	Cell lines were not tested for mycoplasma contamination
Commonly misidentified lines (See ICLAC register)	We did not use any commonly misidentified cell lines.

Flow Cytometry

Plots

Confirm that:

- [ ] The axis labels state the marker and fluorochrome used (e.g. CD4-FITC).
- [ ] The axis scales are clearly visible. Include numbers along axes only for bottom left plot of group (a 'group' is an analysis of identical markers).
- [ ] All plots are contour plots with outliers or pseudocolor plots.
- [ ] A numerical value for number of cells or percentage (with statistics) is provided.

Methodology

Sample preparation	*Describe the sample preparation, detailing the biological source of the cells and any tissue processing steps used.*
Instrument	*Identify the instrument used for data collection, specifying make and model number.*
Software	*Describe the software used to collect and analyze the flow cytometry data. For custom code that has been deposited into a community repository, provide accession details.*
Cell population abundance	*Describe the abundance of the relevant cell populations within post-sort fractions, providing details on the purity of the samples and how it was determined.*
Gating strategy	*Describe the gating strategy used for all relevant experiments, specifying the preliminary FSC/SSC gates of the starting cell population, indicating where boundaries between "positive" and "negative" staining cell populations are defined.*

- [ ] Tick this box to confirm that a figure exemplifying the gating strategy is provided in the Supplementary Information.
